# Encoding of self-initiated actions in axon terminals of the mesocortical pathway

**DOI:** 10.1117/1.NPh.11.3.033408

**Published:** 2024-05-09

**Authors:** Makoto Ohtake, Kenta Abe, Masashi Hasegawa, Takahide Itokazu, Vihashini Selvakumar, Ashley Matunis, Emma Stacy, Emily Froebrich, Nathan Huynh, Haesuk Lee, Yuki Kambe, Tetsuya Yamamoto, Tatsuo K. Sato, Takashi R. Sato

**Affiliations:** a Medical University of South Carolina, Department of Neuroscience, Charleston, South Carolina, United States; b Yokohama City University, Department of Neurosurgery, Yokohama, Japan; c Rutgers, The State University of New Jersey, Robert Wood Johnson Medical School, Center for Advanced Biotechnology and Medicine, Department of Neuroscience and Cell Biology, Piscataway, New Jersey, United States; d Osaka University, Department of Neuro-Medical Science, Osaka, Japan; e College of Charleston, Department of Biology, Charleston, South Carolina, United States; f Kagoshima University, Department of Pharmacology, Kagoshima, Japan; g FOREST, Japan Science and Technology Agency, Saitama, Japan

**Keywords:** two-photon imaging, dopamine, sensory-motor processing, prefrontal cortex

## Abstract

**Significance:**

The initiation of goal-directed actions is a complex process involving the medial prefrontal cortex and dopaminergic inputs through the mesocortical pathway. However, it is unclear what information the mesocortical pathway conveys and how it impacts action initiation. In this study, we unveiled the indispensable role of mesocortical axon terminals in encoding the execution of movements in self-initiated actions.

**Aim:**

To investigate the role of mesocortical axon terminals in encoding the execution of movements in self-initiated actions.

**Approach:**

We designed a lever-press task in which mice internally determine the timing of the press, receiving a larger reward for longer waiting periods.

**Results:**

Our study revealed that self-initiated actions depend on dopaminergic signaling mediated by D2 receptors, whereas sensory-triggered lever-press actions do not involve D2 signaling. Microprism-mediated two-photon calcium imaging further demonstrated ramping activity in mesocortical axon terminals approximately 0.5 s before the self-initiated lever press. Remarkably, the ramping patterns remained consistent whether the mice responded to cues immediately for a smaller reward or held their response for a larger reward.

**Conclusions:**

We conclude that mesocortical dopamine axon terminals encode the timing of self-initiated actions, shedding light on a crucial aspect of the intricate neural mechanisms governing goal-directed behavior.

## 1 Introduction

The prefrontal cortex (PFC) is a pivotal region for various higher cognitive functions.^1^^–^^4^ Proper functioning of the PFC relies on local dopamine released by mesocortical axon terminals originating from neurons in the ventral tegmental area (VTA).^5^^–^^9^ Dysfunction in the mesocortical pathway has been implicated in several psychiatric diseases.^10^^–^^12^ The impact of dopamine on the local circuits of the PFC is mediated via various receptor types, including the D1 receptor and D2 receptor.^6^ Pharmacological and optogenetic studies have suggested distinct roles for D1 and D2 receptors.^7^^,^^8^^,^^13^^,^^14^ For instance, the D1 receptor, but not the D2 receptor, plays a critical role in working memory^15^^–^^19^ and visual attention.^20^ Moreover, in a risk-based decision-making task, infusion of a D1 receptor antagonist reduced risky choices, whereas a D2 receptor antagonist had the opposite effect.^13^

In rodents, the medial PFC (mPFC) coordinates with other areas to initiate goal-directed actions.^21^^–^^25^ The timing of such goal-directed actions is critical to their consequences.^26^ In a situation where a subject reacts to a sensory stimulus, the timing is dictated by sensory and motor processes.^27^^,^^28^ In contrast, in the absence of sensory triggers, the timing of self-initiated actions depends largely on internal states, leading to high variability.^26^^,^^29^ Previous studies, both preclinical and clinical, have shown that systemic administration of dopamine agonists or antagonists influences the timing of action initiation.^30^^–^^32^ However, it remains unclear whether and how the mesocortical pathway contributes to this cognitive process. Given the functional and genetic diversity of dopamine neurons,^33^^–^^35^ encoding various properties such as positive and negative reward prediction errors,^33^^,^^36^^–^^38^ motivation,^39^ timing-related information,^40^ locomotion,^41^^–^^44^ and motor planning/execution,^34^^,^^45^^–^^49^ it is crucial to identify the information conveyed by the mesocortical pathway and its impact on action initiation.

Despite its importance, studies on the information conveyed by the mesocortical pathway have been scarce. Previous studies have measured dopamine concentration in the mPFC^50^^–^^57^ or one-photon gross calcium signals from the mesocortical projections,^58^^,^^59^ neither of which offers high-resolution information on individual axon terminals. One study used antidromic stimulation to identify VTA neurons projecting to the mPFC and examined their responses to noxious stimulation under anesthetized conditions.^60^ Recently, we developed a novel approach based on prism-mediated two-photon imaging *in vivo*, making it possible to visualize axon terminals in the mPFC that originate from the VTA.^39^ In this study, we employed this approach in mice performing a self-timed lever-press task—one type of self-initiated action where mice decide when to press the lever following the onset of an auditory cue, with a longer waiting period resulting in a larger amount of reward. We found that dopaminergic signals mediated via the D2 receptor play critical roles in determining the timing of self-initiated movements, exhibiting ramping activity immediately before action initiation.

## 2 Materials and Methods

### 2.1 Animals

All experimental procedures were approved by the Medical University of South Carolina and Kagoshima University. C57BL/6 mice and eight heterozygous dopamine transporter (DAT)-Cre mice (${\mathrm{Slc}6a3}^{\mathrm{tm}1.1(\mathrm{cre})\mathrm{Bkmn}}$, Jackson Laboratory, #006660, crossed with wild-type C57BL/6) were used in this study.^39^ Mice of both sexes, aged >8 weeks, were included. The mice were maintained in group housing (up to five mice per cage) and experiments were performed during the dark period of a 12-h light/12-h dark cycle.

### 2.2 Headplate Implant and Virus Injection

All surgical procedures were performed aseptically, with the mice under anesthesia with isoflurane. Lidocaine (subcutaneously at the incision) and caprofen (5  mg/kg, intraperitoneally) were applied to prevent pain and brain edema. After surgery, the mice were allowed to recover for at least three days. No experimenter blinding was done.

A custom-made headpost was glued and cemented to the skull,^39^^,^^61^^–^^63^ and then a small craniotomy (<0.5  mm) was performed over the VTA (∼2.9 to 3.5 mm posterior and ∼0.5  mm lateral from the bregma). Inside the small craniotomy, axon-GCaMP virus^64^ (AAV2/1-hSynapsin1-FLEx-axon-jGCaMP8m-WPRE-SV40) was volume-injected to the VTA through a pulled capillary glass (40 to 60  nL/site; depth: 4200 to 4400  μm; 15  min/injection),^61^^–^^63^ as described previously.^39^ After the injection, the craniotomy was sealed with a small piece of cover glass and silicon sealant (Kwik-Cast) and the animals were returned to their home cage.

### 2.3 Behavioral Training in Sensory-Triggered and Self-Timed Lever-Press Tasks

**Fig. 1:**
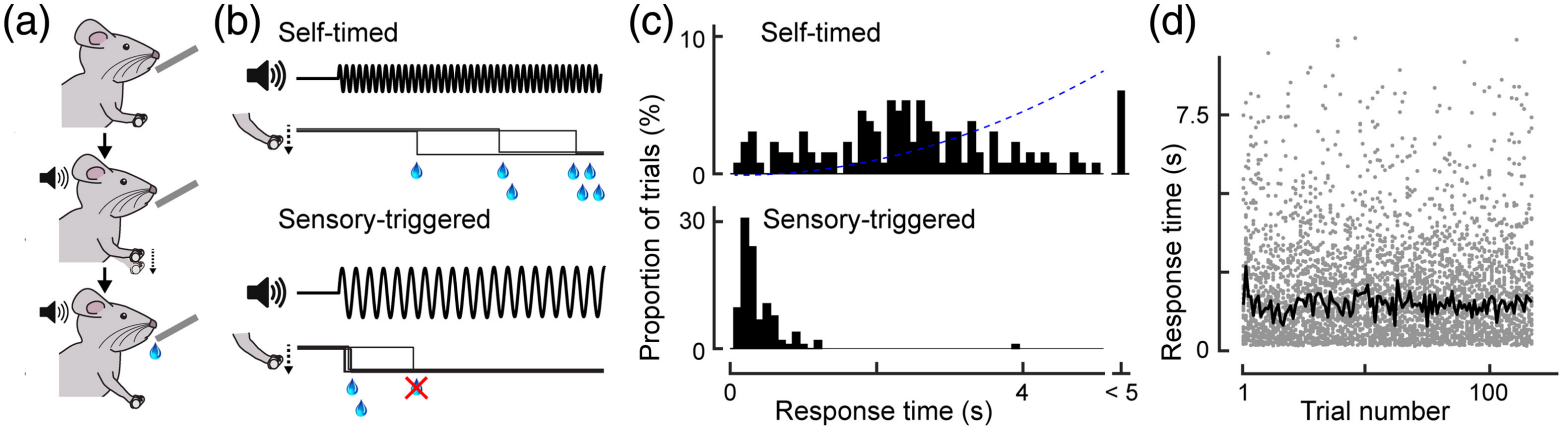
Schematic of the behavioral paradigm. (a) The mice receive a drop of liquid as a reward by pressing the lever. The training on the self-initiated lever-press task followed the training on the sensory-triggered lever-press task. (b) Temporal structures of the tasks. (Top) In the self-initiated lever-press task, the mice receive a high-frequency tone as a warning but without an explicit Go cue. The mice decide when to press the lever. (Bottom) In the sensory-triggered lever-press task, the mice press the lever when a 9 Hz sound is presented. (c) Distribution of the response time in the self-initiated task (top, n=132 trials) and the control task (bottom, n=104 trials) from a single mouse. Blue dashed line indicates the relative amount of reward in relation to the response time. (d) The response time for each trial in the self-initiated lever-press (n=36). The black line indicates the median response time for each trial number across 36 mice. There was no correlation between the median response time and the trial number (p>0.94). The 36 mice are the same as those listed in Tables 1 and 2 in the Supplementary Material. Both the control sessions and the imaging sessions were included in this analysis.

After headpost implantation, mice were trained to perform a sensory-triggered lever-press task with the right forepaw (Fig. 1). Following the lever touch by the mice, after 0.5 to 2.0 s (randomized), a 9 kHz tone with a sound intensity of 70 to 75 dB was presented as a Go cue. The mice were required to press the lever within 1 s to obtain a liquid reward (sucrose water). Lever presses with longer response times were variably rewarded to maintain the motivation of the mice. If the mice released or pressed the lever before the Go cue, the trial was considered as an error. The inter-trial interval was 3 to 6 s. Once the mice learned the sensory-triggered lever-press task, we performed either window implantation together with microprism insertion (see above) or the pharmacological experiments (dopamine antagonist injections). Then, all the mice were trained for the self-timed lever-press task.

After 1 to 2 weeks of sessions with the sensory-triggered task, we trained the mice on the self-timed lever-press task. In this task, when the mice touched the lever, a 14 kHz tone was presented as a warning cue. However, unlike the sensory-triggered lever-press task, the mice decided when to press the lever without a sensory instruction (i.e., without a Go cue). To encourage the mice to delay their response, we rewarded a longer response time with a larger amount of liquid reward. The relationship between the response time and the amount of liquid reward was supra-linear, with the optimum strategy involving waiting >5  s. We conducted one experimental session per day.

### 2.4 Microprism Implant

Once the mice learned the sensory-triggered lever-press task (see above), a microprism^65^^,^^66^ was inserted for two-photon imaging as described previously.^39^ A rectangular craniotomy (4×2  mm) was performed over the bilateral PFC (∼1.5 to 3.5 mm anterior from the bregma), and the dura was removed over the right hemisphere. Then, a microprism implant assembly was inserted into the subdural space within the fissure. The microprism was centered ∼2.5  mm anterior to the bregma to avoid damaging the bridging veins. Once implanted, the prism sat flush against the opposing fissure wall, which contained the medial wall of the PFC (mainly the prelimbic area) in the left hemisphere. The front face of the prism was oriented along the midline.

The assembly consisted of a right-angle microprism (2×2×1  mm, Prism RA N-BK7, Tower Optical Corp.) and two coverslip layers (bottom layer: 4.5×3.0  mm, top layer: 3.6×1.8  mm), which were glued by ultraviolet curing optical adhesive (Norland #81). The top layer of the glass was cemented to the skull with dental acrylic. The imaging was conducted on the mPFC of the left hemisphere, contralateral to the right forepaw used in the lever-press task.

### 2.5 Pharmacological Experiments

Once the mice learned the sensory-triggered and/or self-timed lever-press task, dopamine antagonists were injected into the mPFC. A small hole was made in the skull over the bilateral PFC (∼1.5 to 3.5 mm anterior from the bregma) and covered with silicon sealant (Kwik-Cast). On the day of experiments, the mice were lightly anesthetized with isoflurane and dopamine antagonist (SCH23390,^67^ 5  μg/μl, 100 nl for the D1 receptor antagonist; eticlopride,^8^ 5  μg/μl, 100 nl for the D2 receptor antagonist) or phosphate-buffered saline (PBS) for control experiments was volume-injected (MO-10, Narishige) at a depth of 1.5 mm from the surface. The procedures took place for ∼10  min. After the injection, the craniotomy was sealed with a small piece of cover glass and silicon sealant (Kwik-Cast) and the mice started the behavioral session. We conducted one experimental session per day. Each mouse received either the D1 or D2 receptor antagonist but not both. The control experiments and dopamine antagonist injections were conducted on different days, following a randomized order. The sequence of exposures is described in Table 1 in the Supplementary Material.

### 2.6 In Vivo Two-Photon Calcium Imaging

*In vivo* two photon imaging was performed using a table-mounted microscope (MOM, Sutter Instruments) controlled by ScanImage.^68^ The design of the microscope and the details of the analysis are described in our previous publications.^61^^–^^63^ The light source was a pulsed Ti:sapphire laser (Chameleon Ultra II, Coherent), with the laser wavelength set to 980 nm, which causes a higher fluorescent change in the GCaMP signal and less scattering in the tissue than 920 nm.^61^^,^^63^ The laser power at the apochromatic objective lens (16×, 0.80 NA, Nikon) was <70  mW, and we saw no bleaching. The imaging frame consisted of 512×512  pixels and the frame rate was approximately 30 Hz. For each imaging session, trials with response times of less than 1 s were excluded, and then sorted into three groups with equal numbers based on the response time of the mouse.^28^ We refer to the three groups as the short response time (RT) group, middle RT group, and long RT group.

The imaging data were analyzed similarly to our previous publications^61^^,^^63^. To construct the heatmap shown in Fig. 3(d), the mean activity for each RT group was normalized relative to the maximum baseline activity (3.3 to 0 s before the cue onset) calculated from all the trials. The traces were transformed into percent signal change (ΔF/F), with the baseline for each axon defined as the 30th percentile value of all frames within a 90 s interval. The onsets of the activity for individual axons were determined as the last frame where the activity was below the baseline. The baseline was determined as the 1 s window around the time of the cue onset.

### 2.7 Data Analysis

Data are described as the mean ± s.e.m. unless otherwise noted. The statistical significance for behavioral analysis was determined by the Wilcoxon signed-rank test using MATLAB. Differences in neural activity were determined by the Wilcoxon signed-rank test.

## 3 Results

To investigate the roles of the mesocortical pathway in action initiation, we developed a novel behavioral paradigm for mice: a self-timed lever-press task [Figs. 1(a) and 1(b)]. Each trial began with a warning cue (14 kHz) that signaled the start of the trial. During the trial, the mice decided by themselves when to press the lever; the longer they waited, the larger amount of reward they received. We did not provide sensory instructions as to when to initiate the actions. Although the self-timed lever-press task resulted in a larger variance of response time [Fig. 1(c) top], there was no correlation between the trial number and the response time [Fig. 1(d)]. For each trial number, the median response time across 36 mice remained consistent during the session, and there was no correlation between the trial number and the median response time (p>0.94, Spearman’s rank correlation test, n=36). The overall response time was 1.86±0.07  s (n=36). Before training the self-timed lever-press task, we trained the mice to perform a sensory-triggered lever-press task. In this task, the mice were required to press the lever as soon as a Go cue (9 kHz) was presented [Fig. 1(b)]. The sensory-triggered lever-press task led to a shorter response time (n=36, $p<10^{-8}$ for all mice; Wilcoxon rank sum test) and smaller variance (n=36, p<0.001 for all mice; two-sample F-test for equal variances) than the self-timed lever-press task [Fig. 1(c)]. This result suggests that these movements are triggered by the sensory stimulus, with the timing of action initiation mostly dictated by sensory and motor processes.^27^^,^^28^.

**Fig. 2:**
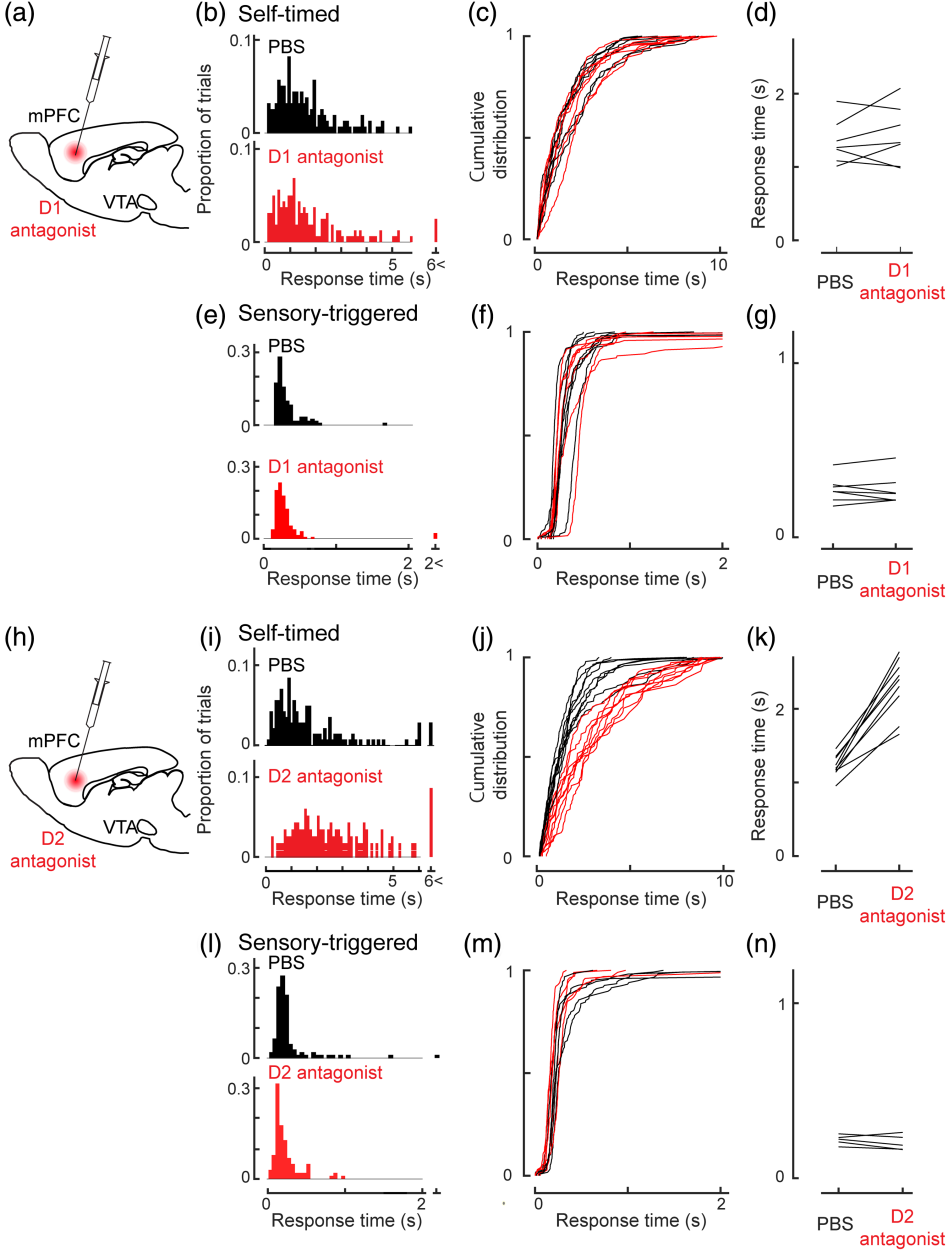
Effects of dopamine antagonists on task performance. (a) Experimental design for D1 antagonist injection. (b) Distributions of response times from one example mouse in the self-timed lever-press task following D1 antagonist injection. Black, PBS injection, n=159 trials. Red, D1 antagonist injection, n=160 trials (p>0.70). (c) Empirical cumulative distribution of the response time in the self-timed lever-press task for seven mice. PBS injection is shown in black and D1 antagonist injection is shown in red. The numbers of trials were 136, 114, 105, 143, 122, 159, and 115 for PBS injection and 133, 116, 103, 157, 125, 160, and 127 for D1 antagonist injection. (d) Across the population of seven mice, the D2 antagonist injection did not affect the median response time in the self-timed lever-press task (p>0.50, n=7 mice). (e) Distributions of response times from one example mouse in the self-timed lever-press task following D1 antagonist injection. Black, PBS injection, n=117 trials. Red, D1 antagonist injection, n=123 trials (p>0.15). (f) Empirical cumulative distribution of the response time in the sensory-triggered lever-press task. PBS injection is shown in black and D1 antagonist injection is shown in red. The numbers of trials were 121, 127, 111, 117, 120, 106, and 112 for PBS injection and 108, 121, 104, 123, 120, 103, and 108 for D1 antagonist injection. (g) Across the population of 7 mice, the median response time was not affected (p>0.80, n=7). (h) Experimental design for D2 antagonist injection. (i) Distributions of response times from one example mouse in the self-timed lever-press task following D2 antagonist injection. Black, following PBS injection, n=143 trials. Red, following D1 antagonist injection, n=116 trials. The response time was longer following D2 antagonist injection (p<0.001). (j) Empirical cumulative distribution of the response time in the self-timed lever-press task. PBS injection is shown in black and D1 antagonist injection is shown in red. The numbers of trials were 112, 144, 134, 141, 122, 143, 143, 143, and 147 for PBS injection and 87, 29, 105, 113, 101, 92, 116, 90, and 109 for D2 antagonist injection. (k) Across the population of nine mice, the D2 antagonist injection resulted in a longer response time with a reduced number of immediate lever presses in the self-timed task (p<0.004, n=9 mice). (l). Distributions of response times from one example mouse in the sensory-triggered lever-press task following D2 antagonist injection. Black, PBS injection, n=110; Red, D2 antagonist, n=101 (p>0.37). (m) Empirical cumulative distribution of the response time in the sensory-triggered lever-press task. PBS injection is shown in black and D2 antagonist injection is shown in red. The numbers of trials were 118, 110, 124, 123, and 127 for PBS injection and 108, 101, 99, 98, and 115 for D2 antagonist injection. (n) Across the five mice, injection of a D2 antagonist did not affect performance in the sensory-triggered lever-press task (p>0.31, n=5).

We next examined the significance of the dopamine input to the mPFC in our self-timed lever-press task and sensory-triggered lever-press task by pharmacological experiments with dopamine antagonists [Figs. 2(a) and 2(h)]. Injection of a D1 antagonist (SCH23390, 5  μg/μl, 100 nl) into the mPFC had no effect on the self-timed lever-press task (n=7; p>0.50; Wilcoxon signed-rank test) [Figs. 2(b)–2(d)]. However, when we injected a D2 antagonist (eticlopride, 5  μg/μl, 100 nl), the response time in the self-timed lever-press task substantially increased (n=9; p<0.004; Wilcoxon signed-rank test) [Figs. 2(i)–2(k)]. The effect of the D2 antagonist did not persist on the following day (Fig. S2-1 in the Supplementary Material). Additionally, we compared the response times of the first 50 trials of the sessions between the D2 antagonist and control experiments, and the difference persisted (Fig. S2-2 in the Supplementary Material). We injected the D2 antagonist into a neighboring area, the medial orbitofrontal cortex, which did not affect the response time (Fig. S2-3 in the Supplementary Material, p>0.90). This result demonstrated the spatial specificity of the role of D2 receptor in the self-timed lever-press task. Injection of either the D1 or D2 antagonist did not affect the animal’s performance in the control, sensory-triggered lever-press task (D1 antagonist, p>0.80, n=7; D2 antagonist, p>0.31, n=5; Wilcoxon signed-rank test) [Figs. 2(e)–2(g) for D1 antagonist; Figs. 2(l)–2(n) for D2 antagonist]. Thus, mesocortical projections to the mPFC play critical roles in self-timed actions via the D2 receptor.

**Fig. 3:**
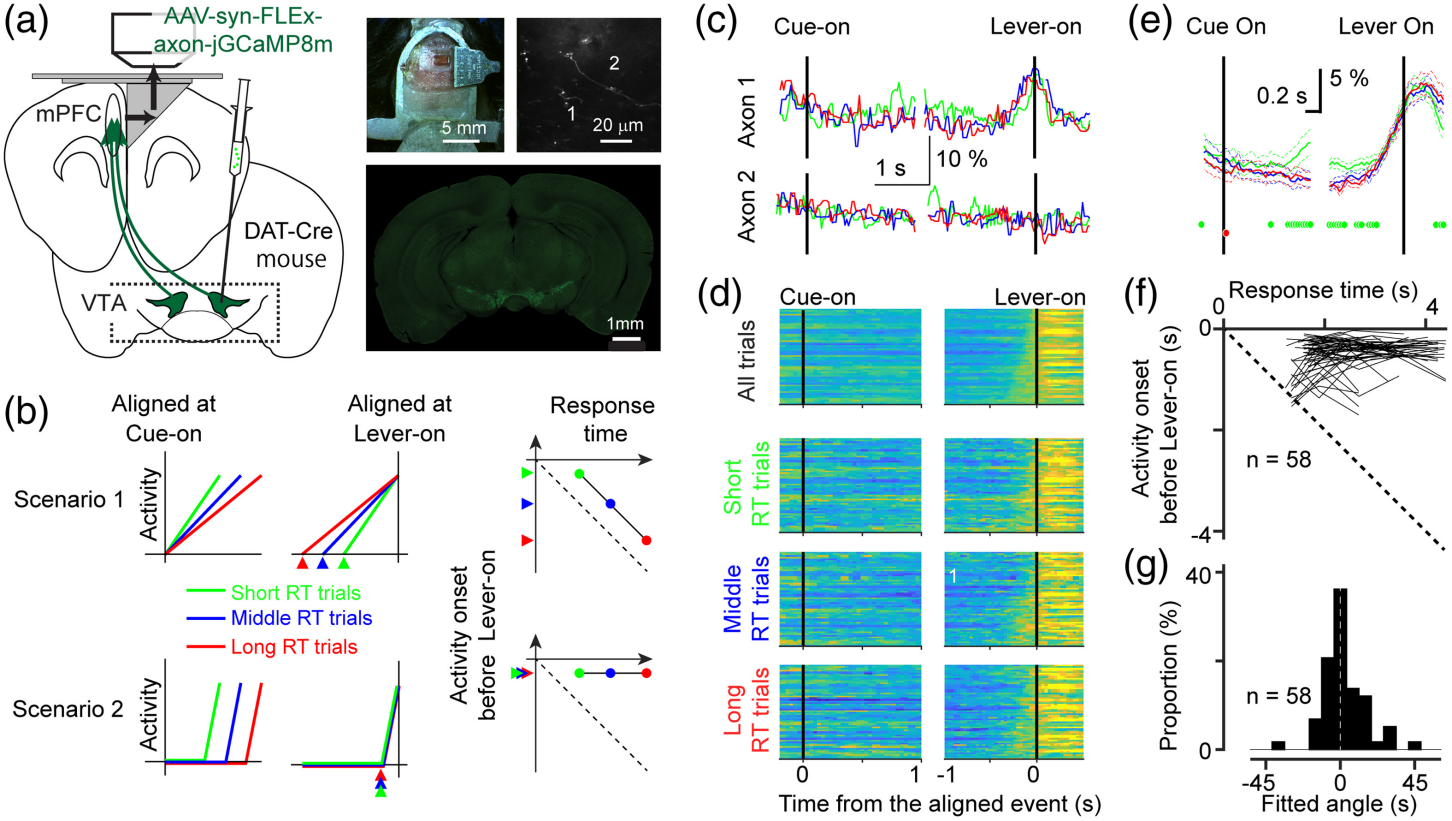
Activity of mesocortical axon terminals during the self-initiated lever-press task. (a) Experimental design. (Left) Virus expressing jGCaMP8m was injected to the VTA and the axon terminals in the mPFC were imaged via two -photon microscopy. (Right, top) Dorsal view of a prism-implanted animal and a two-photon image of axons inside the prism. (Right, bottom) A coronal section showing jGCaMP8m expression in the VTA. (b) Hypothetical activity patterns during the self-initiated lever-press task. Trials are sorted into three groups based on the response time:^69^^,^^70^ the short RT group (green), middle RT group (blue), and long RT group (red). If the neural activity encodes motor command, the activity would increase at a fixed time before the lever press regardless of the response time (top). In this case, the relationship between the onset of the activity (before the lever press) and the response time would be −45  deg in the two-dimensional plot (right, top). If the neural activity encodes motor preparation, the activity would increase gradually with a different slope until the movement onset (bottom). In this case, the relationship would be 0 deg (right, bottom).^71^ (c) Activity of two axon terminals during the self-initiated lever-press task. (d) Activity of all the axon terminals that exhibited increased activity before the lever press (n=58). The activity patterns were similar whether the response time was short, middle, or long. (e, f) The mean activity of all the axon terminals that showed increased activity before the lever press (n=58) aligned by cue onset (left) and lever onset (right). The green dots indicate the frame where the activity in the short RT and middle RT groups was significantly different (p<0.05). The red dot indicates the frame where the activity in the long RT and middle RT group was significantly different (p<0.05). (f) The relationship between the activity onset and the response time of the mouse for the short RT, middle RT, and long RT groups for each neuron (n=58). In the majority of neurons, the time between the activity onset and the lever press was fixed. (g) The distribution of the regression slope for individual axons in panel (f). The values were clustered at 0 degrees (compared to 0 deg, p=0.15; compared to −45  deg, p<0.0001). We also conducted the analyses in panels (d)–(g) by grouping trials based on consistent time windows, achieving the same results (see Fig. S3-1 in the Supplementary Material).

We next investigated the information conveyed via the mesocortical pathway. We injected Cre-dependent adeno-associated virus into the midbrain regions of transgenic mice (DAT-Cre^57^), which express Cre-recombinase in dopamine neurons^58^ [see Sec. 2 and Fig. 3(a)]. Our previous study confirmed that GCaMP expression in cell bodies in the VTA (and substantia nigra pars compacta) coincides with the expression of tyrosine hydroxylase, an endogenous marker for dopamine neurons.^39^ We imaged the axon terminals of these dopaminergic neurons in the mPFC using *in vivo* two-photon imaging combined with microprism insertion [Fig. 3(a)]. Unlike our previous study, we were unable to administer aversive stimuli^39^ because providing such stimuli would hamper the performance of the mice. Our investigation centered on determining whether mesocortical axon terminals exhibit ramping activity before action initiation and, if so, how this activity correlates with the animals’ response time [Fig. 3(b)].^71^^,^^72^

We specifically explored two possible scenarios regarding the ramping activity of mesocortical dopamine axons. One possibility is that the ramping activity could gradually increase after cue onset in a manner predicting response time, akin to activity patterns observed in higher motor areas^71^^,^^72^ [Fig. 3(b), top], reflecting the accumulation of motor plans or decisions. An alternative possibility is that the ramping activity could be initiated at a fixed time before action initiation [Fig. 3(b), bottom], suggesting that it reflects the execution of the movement. To distinguish between these two possibilities, we analyzed the activity of individual axon terminals in three groups of trials that were sorted based on the response times (green for short RT, blue for middle RT, red for late RT). We identified that a significant proportion of axon terminals exhibited ramping activity before the lever press (n=58 out of 249 axon terminals, 8 mice)^61^ [Figs. 3(c) and 3(d)]. The axon terminal shown in Fig. 3(c) displayed similar activity patterns before the lever press, regardless of the response time length, consistent with scenario 2 in Fig. 3(b). This pattern held true across the population [Fig. 3(c)], and the average across 58 axons exhibited a similar activity pattern toward the execution of the lever press, whether the response time was short, middle, or long [Fig. 3(e)]. Across the population, the difference between the short and middle RT group was non-significant (p<0.01) for the last 330 ms before the lever press, and the difference between the middle and long RT group was non-significant for the last 1.7 s.

Finally, we examined the two possible scenarios regarding how the ramp-up activity of individual axon terminals depends on response time [Fig. 3(b)]. For each axon terminal, we determined the onset time for the short, middle, and long RT groups (see Sec. 2) and plotted them against the response time of the mice in each group. Across trial groups with different response times [Fig. 3(b), right column], we found that the ramp-up time (period between the onset time and the lever press) was constant across trials [Fig. 3(f)]. To quantify this, for each axon, we computed the angle of the regression line that connects the three points corresponding to the short, middle, and long RT groups [Fig. 3(g)]. The circular mean of the angle was close to 0 deg (2.44 deg; difference from 0 deg, p=0.15; difference from −45  deg, p<0.0001). Therefore, we conclude that mesocortical activity initiates at a fixed time before action initiation, thus containing information related to the execution of the movements.

## 4 Discussion and Conclusion

Dopamine projections to the mPFC are recognized as crucial neuromodulators for the proper functioning of the mPFC. Various pharmacological experiments utilizing dopamine receptor blockers in the PFC have consistently induced a range of cognitive deficits.^7^^,^^8^^,^^13^^,^^15^^–^^20^ Despite this, the role of dopamine in the PFC in action initiation is controversial,^67^^,^^69^ and the information encoded by this pathway has remained unclear. Previous studies have proposed that certain dopaminergic neurons may encode action initiation.^34^^,^^46^^,^^70^^,^^73^ For example, the activity of neurons in the substantia nigra pars compacta increases before mice transition from an immobility state to a mobility state.^46^ However, the previous studies did not measure the activity of neurons constituting the mesocortical projections, leaving uncertainty about whether the mesocortical pathway contributes to action initiation. Our study addresses this gap by demonstrating that the mesocortical pathway exhibits pre-movement activity and contributes to action initiation via D2 receptors in self-timed tasks, in contrast to its lack of involvement in sensory-triggered lever-press tasks.

A few studies in primates have implicated the potential roles of D2 receptors in action initiation. Goldman–Rakic and her colleagues’ pioneering work showed that D2 receptors modulate neural activity in the PFC associated with saccades in a memory-guided saccade task.^67^ Another study employing pro- and anti-saccade tasks demonstrated that D2 receptor stimulation selectively modulated eye-movement-related activity.^69^ Notably, in these studies, the timing of action initiation was instructed by a sensory stimulus, and there were no^67^ or minimum^69^ effects of the D2 receptor on response time. In the present study, using a novel mouse behavioral paradigm, we demonstrate substantial effects of D2 receptor blockers in a self-timed lever-press task but not a sensory-triggered lever-press task. Furthermore, the effects were absent following D1 receptor antagonist injection, with an amount similar to that used in previous studies.^74^ However, we cannot completely exclude the possibility that a higher concentration of the D1 antagonist might have some effects. Our study extends the previous reports on the relationship between D2 receptors in the PFC and movement-related activity, suggesting that such activity might play a major role in self-timed actions but not in sensory-triggered actions.

Our behavioral results are further supported by the existence of pre-movement activity of dopaminergic axon terminals in the PFC. Until recently, monitoring the activity of individual dopamine axon terminals in the mPFC was challenging. Our group is the first to accomplish this by combining *in vivo* two-photon imaging and microprism insertion.^39^ Importantly, our approach preserves the local circuit integrity near the imaging regions. Specifically, our mice conducted the lever press with the right forepaw, and our imaging was performed from the left mPFC in order to avoid disrupting the most relevant motor region. Our approach allowed us to uncover that different axon terminals start exhibiting pre-movement activity at different time points before the action.

Interestingly, the activity patterns of individual axon terminals were very similar whether the animals responded immediately for a small amount of reward or waited longer for a larger amount of reward, indicating that the mesocortical pathway does not encode the accumulation of preparation, at least in our behavioral context. This finding provides a striking contrast with a recent study that monitored the bulk activity of the nigrostriatal pathway, where the summed activity of all the axons exhibited ramp activity immediately after the start-time cue in a self-timed task [similar to Fig. 3(b), top].^48^ Further studies will be required to determine whether the discrepancy between our study and the previous one might be explained by (1) the specific dopaminergic pathway (mesocortical versus nigrostriatal), (2) technical differences (individual axons vs. bulk imaging), or (3) differences in the behavioral task (timing determined by the mice in our task vs. being specified by the experimenter in the previous study). Regardless, it is still not entirely clear how such ramping activity is conveyed to the downstream circuits in the mPFC. One attractive possibility is that D2 receptors activate a specific group of layer V neurons that project subcortically.^75^ Alternatively, dopamine might act at D2 receptors in the interneurons to suppress inhibitory transmission.^76^ A promising avenue of research is to visualize the activity of D2 receptor-expressing neurons together with mesocortical axon terminals using calcium sensors of different colors.

The mesocortical pathway has been implicated in various psychiatric disorders,^10^^–^^12^ yet technical challenges have hindered us from fully investigating the information conveyed by this pathway. The techniques we have employed in this study, together with appropriate behavioral paradigms, could lead to a better understanding of these disorders.

## Supplementary Material



## Data Availability

These studies did not generate unique reagents. All data reported in this study will be shared by the corresponding authors upon request. All analysis codes from this study are available from the corresponding authors upon request. Further information and requests for reagents may be directed to the Lead Contact, Takashi R Sato (satot@musc.edu).
