# Supplementary material for: Encoding of self-initiated actions in axon terminals of the mesocortical pathway
Source: Neurophotonics. 2024 May 9;11(3):033408. doi: 10.1117/1.NPh.11.3.033408 (PMC11080647; doi:10.1117/1.NPh.11.3.033408)
Supplement: Supplementary file 1 [file NPh_011_033408_SD001.pdf]

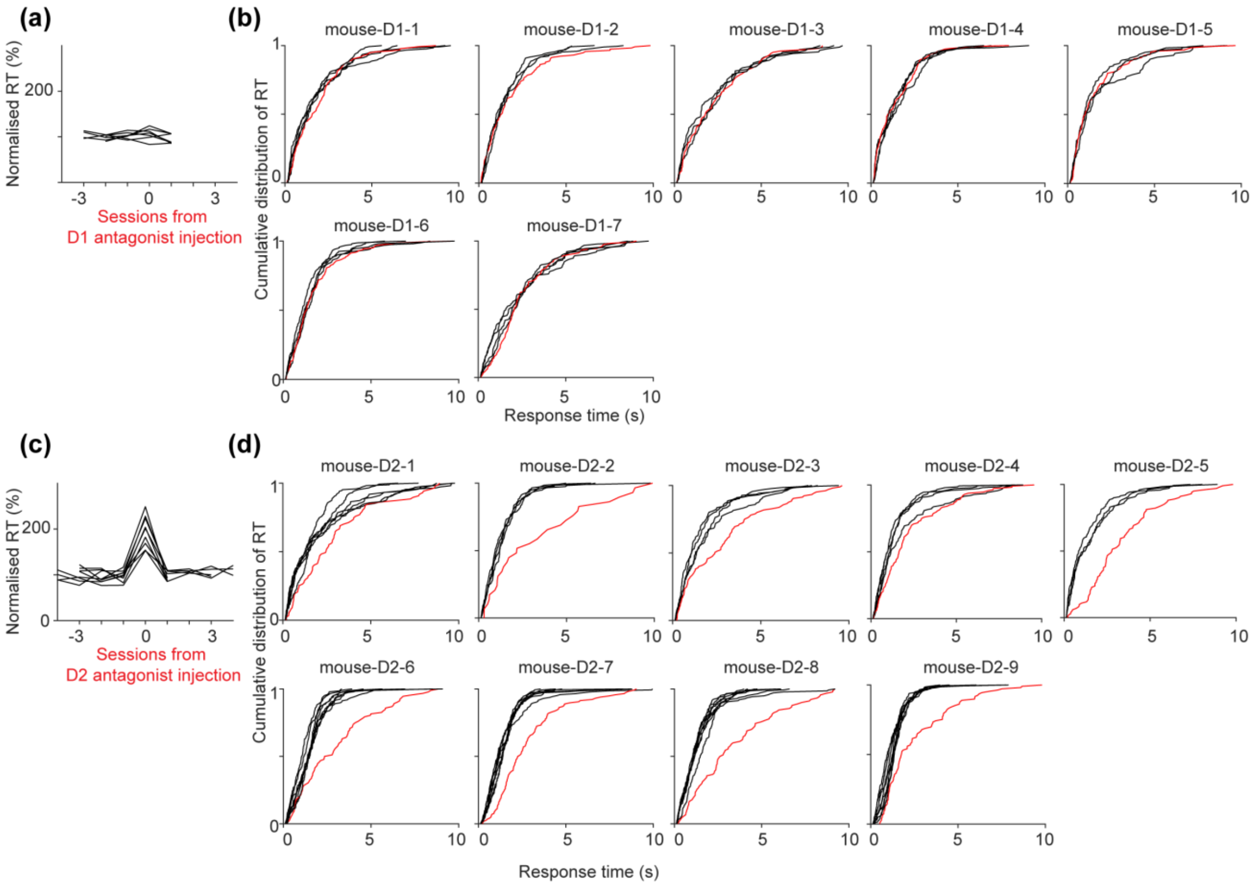

Fig. S2-1: (**a**). The median response time of individual mice before and after the session of D1 antagonist injection (*n* = 7 mice). Each line represents one mouse. (**b**). The empirical cumulative distribution of the response time for each session in each mouse. Number of trials for each mouse are shown below. (**c**). The median response time of individual mice before and after the session of D2 antagonist injection (*n* = 9 mice). Each line represents one mouse. (**d**). The empirical cumulative distribution of the response time for each session in each mouse.

Number of trials
D1-1, PBS, 124, 114, 124, 136; D1 antagonist 133.
D1-2, PBS, 114, 133, 194; D1 antagonist 116
D1-3, PBS, 87, 105, 85; D1 antagonist 103
D1-4, PBS, 124, 143, 131, 147; D1 antagonist 157
D1-5, PBS, 71, 123, 122; D1 antagonist 125
D1-6, PBS, 159, 157, 159, 161; D1 antagonist 160
D1-7, PBS, 112, 129, 95, 115; D1 antagonist 127

D2-1, PBS: 115, 134, 111, 112, 147, 169; D2 antagonist: 87.
D2-2, PBS: 119, 144, 174, 135; D2 antagonist: 29.
D2-3, PBS: 114, 116, 124, 135; D2 antagonist: 105.
D2-4, PBS: 141, 147, 148, 145; D2 antagonist: 113.
D2-5, PBS: 140, 129, 122; D2 antagonist 101.
D2-6, PBS: 144, 145, 143, 144, 143, 137, 147, 146; D2 antagonist 92
D2-7, PBS: 145, 143, 146, 146, 144, 144, 143, 143; D2 antagonist 116
D2-8, PBS: 145, 144, 144, 143, 151, 143, 138, 142; D2 antagonist 90
D2-9, PBS: 146, 144, 149, 147, 144, 145, 144, 143; D2 antagonist 109

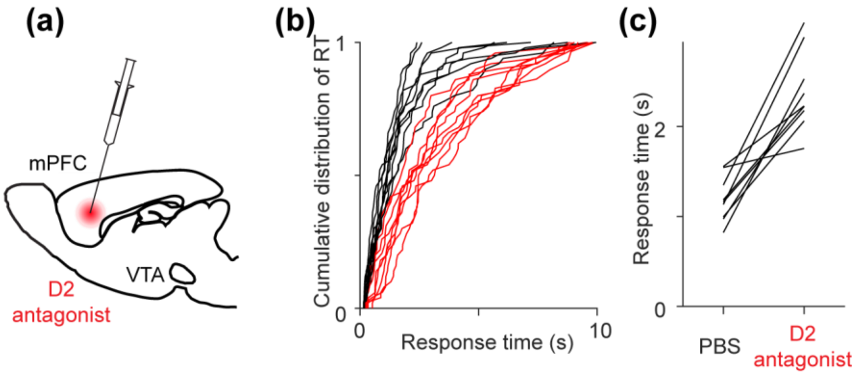

Fig. S2-2: Effects of dopamine antagonists on task performance. Same experiments as Fig. 2h-k, but analysis was based on the initial 50 trials. (a) Experimental design for D2 antagonist injection. (b) Empirical cumulative distribution of the response time in the self-timed lever-press task. PBS injection is shown in black and D1 antagonist injection is shown in red. The number of trials was 50 for all mice, except for one for the D2 antagonist injection (29 trials). (c) In the self-timed lever-press task, the D2 antagonist injection resulted in a longer response time in the initial 50 trials ($p < 0.004$, $n = 9$ mice; one mouse performed only 29 trials in the D2 antagonist session).

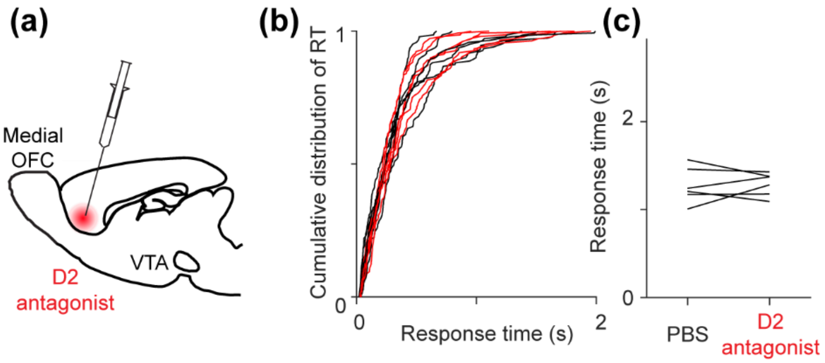

Fig. S2-3: Effects of D2 antagonist injection in the medial orbitofrontal cortex on task performance. (a) Experimental design for D2 antagonist injection. (b) Empirical cumulative distribution of the response time in the self-timed lever-press task. PBS injection is shown in black and D2 antagonist injection is shown in red. The numbers of trials were 151, 131, 143, 143, 143, and 143 for PBS injection and 149, 144, 136, 137, 143, and 145 for D2 antagonist injection. (c) The D2 antagonist injection did not affect the response times ($p < 0.99$, $n = 6$ mice).

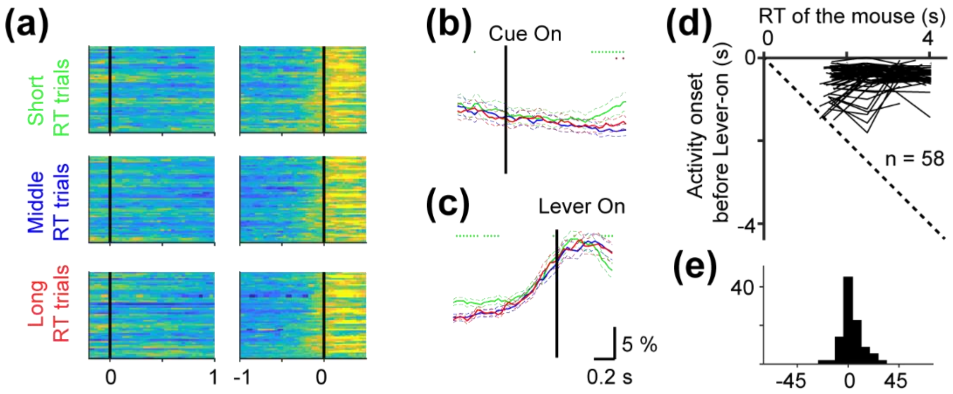

Fig. S3-1: Activity of mesocortical axon terminals during the self-initiated lever-press task. We used 1-2s, 2-3s, and 3s- for the short, middle, and long RT groups instead of the 33rd and 67th percentiles. The same conventions are used as in Fig. 3 d, e, f, and g. In e, the values were clustered at 0 degrees (compared to 0º, $p = 0.36$; compared to -45º, $p < 0.0001$).

Mouse Name	Sex	Injection site	Experiment	Sensory-trigger	Self-timed
Mouse D1-1	M	mPFC	D1R antagonist	D1A → PBS	D1A → PBS
Mouse D1-2	M	mPFC	D1R antagonist	D1A → PBS	PBS → D1A
Mouse D1-3	F	mPFC	D1R antagonist	PBS → D1A	PBS→ D1A
Mouse D1-4	F	mPFC	D1R antagonist	PBS → D1A	PBS→ D1A
Mouse D1-5	M	mPFC	D1R antagonist	PBS → D1A	D1A → PBS
Mouse D1-6	M	mPFC	D1R antagonist	PBS → D1A	PBS → D1A
Mouse D1-7	F	mPFC	D1R antagonist	D1A → PBS	D1A → PBS
Mouse D2-1	M	mPFC	D2R antagonist	D2A → PBS	D2A → PBS
Mouse D2-2	M	mPFC	D2R antagonist	D2A → PBS	PBS → D2A
Mouse D2-3	F	mPFC	D2R antagonist	D2A →PBS	D2A → PBS
Mouse D2-4	F	mPFC	D2R antagonist	PBS →D2A	PBS → D2A
Mouse D2-5	M	mPFC	D2R antagonist	PBS → D2A	D2A → PBS
Mouse D2-6	F	mPFC	D2R antagonist	NA	PBS → D2A
Mouse D2-7	M	mPFC	D2R antagonist	NA	PBS → D2A
Mouse D2-8	M	mPFC	D2R antagonist	NA	PBS → D2A
Mouse D2-9	F	mPFC	D2R antagonist	NA	PBS → D2A
Mouse MO-1	M	mOFC	D2R antagonist	NA	D2A → PBS
Mouse MO-2	M	mOFC	D2R antagonist	NA	D2A → PBS
Mouse MO-3	M	mOFC	D2R antagonist	NA	D2A → PBS
Mouse MO-4	F	mOFC	D2R antagonist	NA	PBS→ D2A
Mouse MO-5	F	mOFC	D2R antagonist	NA	PBS→ D2A
Mouse MO-6	M	mOFC	D2R antagonist	NA	PBS→D2A

Supplementary Table 1. List of mice used for pharmacological experiments (Fig. 2, Fig. S2-1, S2-2, S2-3). The numbers of trials are listed in the legends of the individual figures.

Mouse Name	Sex	Session ID	N of movement-related axons
Mouse VTA-1	M	1	2
		2	1
		3	2
Mouse VTA-2	M	1	1
Mouse VTA-3	F	1	4
Mouse VTA-4	F	1	3
Mouse VTA-5	M	1	4
		2	3
		3	1
Mouse VTA-6	M	1	1
Mouse VTA-7	F	1	1
		2	1
Mouse VTA-8	M	1	4
Mouse VTA-9	M	1	4
Mouse VTA-10	F	1	6
Mouse VTA-11	F	1	2
Mouse VTA-12	M	1	9
Mouse VTA-13	M	1	6
Mouse VTA-14	M	1	3

Supplementary Table 2. List of mice used for two-photon imaging experiments (Fig. 3).
